# Myeloid ZFP36L1 Does Not Regulate Inflammation or Host Defense in Mouse Models of Acute Bacterial Infection

**DOI:** 10.1371/journal.pone.0109072

**Published:** 2014-10-09

**Authors:** Lynnae D. Hyatt, Gregory A. Wasserman, Yoon J. Rah, Kori Y. Matsuura, Fadie T. Coleman, Kristie L. Hilliard, Zachary Ash Pepper-Cunningham, Michael Ieong, Deborah J. Stumpo, Perry J. Blackshear, Lee J. Quinton, Joseph P. Mizgerd, Matthew R. Jones

**Affiliations:** 1 Pulmonary Center, Boston University School of Medicine, Boston, Massachusetts, United States of America; 2 Department of Medicine, Boston University School of Medicine, Boston, Massachusetts, United States of America; 3 Department of Microbiology, Boston University School of Medicine, Boston, Massachusetts, United States of America; 4 Laboratory of Signal Transduction, National Institute of Environmental Health Sciences, National Institutes of Health, Research Triangle Park, North Carolina, United States of America; 5 Departments of Medicine and Biochemistry, Duke University Medical Center, Durham, North Carolina, United States of America; 6 Department of Pathology and Laboratory Medicine, Boston University School of Medicine, Boston, Massachusetts, United States of America; 7 Department of Biochemistry, Boston University School of Medicine, Boston, Massachusetts, United States of America; French National Centre for Scientific Research, France

## Abstract

Zinc finger protein 36, C3H type-like 1 (ZFP36L1) is one of several Zinc Finger Protein 36 (Zfp36) family members, which bind AU rich elements within 3′ untranslated regions (UTRs) to negatively regulate the post-transcriptional expression of targeted mRNAs. The prototypical member of the family, Tristetraprolin (TTP or ZFP36), has been well-studied in the context of inflammation and plays an important role in repressing pro-inflammatory transcripts such as TNF-α. Much less is known about the other family members, and none have been studied in the context of infection. Using macrophage cell lines and primary alveolar macrophages we demonstrated that, like ZFP36, ZFP36L1 is prominently induced by infection. To test our hypothesis that macrophage production of ZFP36L1 is necessary for regulation of the inflammatory response of the lung during pneumonia, we generated mice with a myeloid-specific deficiency of ZFP36L1. Surprisingly, we found that myeloid deficiency of ZFP36L1 did not result in alteration of lung cytokine production after infection, altered clearance of bacteria, or increased inflammatory lung injury. Although alveolar macrophages are critical components of the innate defense against respiratory pathogens, we concluded that myeloid ZFP36L1 is not essential for appropriate responses to bacteria in the lungs. Based on studies conducted with myeloid-deficient ZFP36 mice, our data indicate that, of the Zfp36 family, ZFP36 is the predominant negative regulator of cytokine expression in macrophages. In conclusion, these results imply that myeloid ZFP36 may fully compensate for loss of ZFP36L1 or that *Zfp36l1*-dependent mRNA expression does not play an integral role in the host defense against bacterial pneumonia.

## Introduction

Pneumonia is well-recognized to be a pervasive and significant public health concern [1], [2]. Although the lung mounts strong innate defenses to localize and eliminate pathogenic bacteria [2]–[6], that same inflammatory response can also cause severe local and systemic damage. Uninhibited, excessive pulmonary inflammation can lead to a syndrome of diffuse alveolar epithelial damage and capillary leakage resulting in acute lung injury (ALI) [7]–[9]. Thus, intricate mechanisms of gene regulation are needed to orchestrate the delicate balance between mounting effective host defenses while minimizing lung injury.

**Figure 1:**
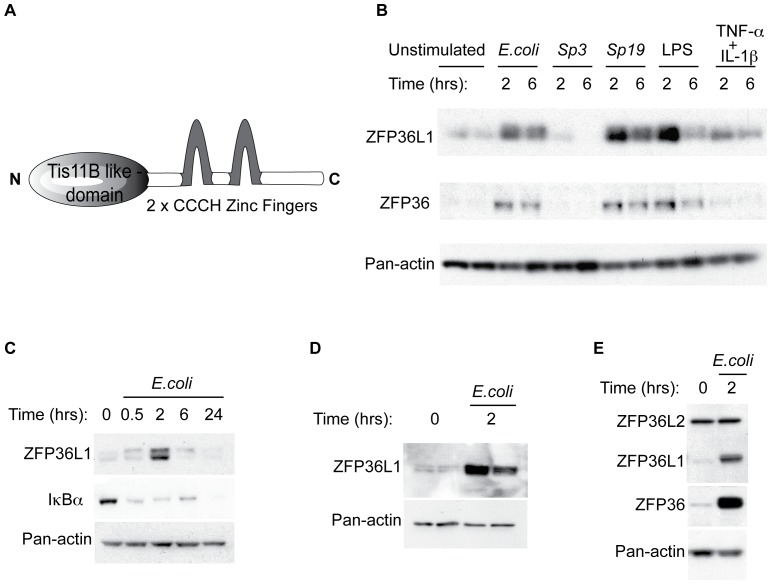
ZFP36L1 is induced in macrophages in response to infectious and inflammatory stimuli. (A) Graphic representation of Zfp36 proteins showing TIS11B like domain and two tandem zinc fingers, which facilitate binding to RNA. Immunoblot analysis of protein derived from (B) Alveolar macrophage derived cells (MH-S cell line) stimulated *in vitro* with *E. coli*, *S. pneumoniae* serotype 3 or 19, LPS, or a combination TNF- α and IL-1β. (C) MH-S cells during a time course of *E. coli* infection (D) Primary murine alveolar macrophages stimulated *in vitro* with *E. coli* or (E) Primary human alveolar macrophages stimulated *in vitro* with *E. coli*. Immunoblot results are representative of three independent experiments.

In addition to transcriptional activation of host defense genes during infection, fine-tuning of a dynamic inflammatory response requires a further post-transcriptional level of gene regulation. Once initiated, the cessation of pro-inflammatory cytokine transcription does little to halt or regulate the level of cytokine protein output, which may continue even after transcription has stopped [10], [11]. mRNA sequence *cis* elements known to negatively regulate cytokine transcript translation are adenine and uridine rich elements (AREs) which, when bound by ARE-binding proteins such as the Zfp36 family members, mediate translation, degradation, and subcellular localization [12]–[14]. Zfp36 family members contain two tandem zinc finger domains (CCCH) that bind to the ARE to initiate deadenylation and subsequent decay (Figure 1A) [15]–[19]. This family is comprised of four members including *Zfp36* (Tristetraprolin, TTP), *Zfp36l1* (butyrate response factor 1, Brf1), and *Zfp36l2* (butyrate response factor 2, Brf2). These proteins are highly conserved across species, with an 87% sequence identity between the mouse and human sequences for *Zfp36* [20]. The fourth family member present in rodents but not in humans, *Zfp36l3*, has been identified in placental tissue and in low levels in adipocytes [21], [22]. Originally investigated as potential transcription factors, these genes are rapidly induced in response to insulin and growth factors with subsequent translocation from the nucleus to the cytosol [20], [23]–[28]. Their importance in regulation of inflammatory mediators was later identified through gene knockout studies in mice [15], [19], [29], [30]. While these proteins have>70% amino acid identity in their DNA-binding zinc finger regions, there is evidence to suggest that they have differential expression in different tissues, and interact with both overlapping and unique transcripts [31]–[33].

The most well characterized family member, ZFP36 has been shown to interact with more than one hundred different mRNAs including many cytokines and chemokines [34], and plays an important immune regulatory role [10]. Mice deficient in ZFP36 consistently develop a systemic inflammatory syndrome with cachexia, polyarthritis, dermatitis, and inflammation of nearly every organ [29]. This syndrome is largely due to elevated circulating levels of TNF- α at baseline. It can be reproduced with a model of TNF- α overproduction [35], [36], and rescued with anti-TNF-α antibodies [29], and mice lacking TNF-α receptors are protected from this syndrome [37]. Furthermore, the post-transcriptional regulation of TNF-α was demonstrated when mice engineered to lack the TNF- α ARE developed a similar but more severe phenotype to that of the ZFP36 deficient mice [38], providing further evidence that the phenotype is due to the interaction of ZFP36 with the ARE. Myeloid cells, particularly macrophages, have been implicated as the major source of TNF overproduction in these mice [15], [39]. However, while myeloid specific deletion of ZFP36 results in an increase in macrophage-derived TNF-α following LPS stimulation, the phenotype is apparent only with stimulation and not at baseline [40].

In contrast to ZFP36-deficient mice which survive until birth, germ line deletion of ZFP36L1 is embryonic lethal [41]. Further supporting unique specific roles for this family member, recent work has determined that ZFP36L1 is involved in maintaining the pluripotency of mESCs [42] and is a negative regulator of plasmacytoid differentiation [43]. However, other studies and settings provide evidence for functional redundancy in this family. For example, the mutation of both ZFP36L1 and ZFP36L2 together, but not of either family member alone, disrupts thymic development of T cells and leads to acute lymphoblastic leukemia [44]. Multiple mRNA targets have been identified for ZFP36L1, but there are discrepancies amongst studies and little consensus about which transcripts are directly influenced by this protein. Some targets identified from cell culture and overexpression systems have been termed “non-physiological” targets, as compared to others identified from knockout animals (termed “physiological”) [45]. Given the important role that ZFP36 proteins play in the regulation of inflammation, along with the apparent importance in macrophages for their functioning, we sought to determine whether myeloid deficiency of ZFP36L1 would have an impact on host defense and lung injury during acute bacterial pneumonia. Several of the proposed targets of ZFP36L1 (both physiological and non-physiological) include VEGF, TNF- α, GM-CSF and IL-3, all which have a defined role in the inflammatory response of the lung. [18], [46]–[50] We found that ZFP36L1 production is induced in alveolar macrophages during pneumonia, and we hypothesized that this induction serves to limit pro-inflammatory cytokine mediated lung injury. Furthermore, we hypothesized that a myeloid-specific deficiency of ZFP36L1 would result in elevated levels of lung cytokines and therefore increased efficiency of host defense against pneumonia, but at the cost of increased lung damage. Surprisingly, our investigations demonstrate that, despite its increased production by macrophages in response to pneumonia, myeloid ZFP36L1 alone is not essential for the lung's defense against pneumonia.

## Materials and Methods

### Ethics Statement

All human studies were approved by the Institutional Review Board (IRB) of Boston University Medical Center. Human samples were obtained only after informed written consent. Mouse experiments were performed in accordance with US Federal Law and approved by the Boston University School of Medicine Institutional Animal Care and Use Committee (IACUC) (Permit # 14859). Experiments were performed under approved anesthesia, and all efforts were made to minimize suffering.

### Cell culture

The murine alveolar macrophage-derived MH-S cell line was obtained from the American Type Culture Collection (ATCC), seeded in 6 or 12 well culture plates, and incubated overnight at 37°C in a humidified atmosphere containing 5% CO_2_. The cells were stimulated with 10^6^ colony forming units (CFU) *Escherichia coli* (*E. coli*, ATCC-19138), *Streptococcus pneumoniae* serotype 3 (Sp3; ATCC-6303), *Streptococcus pneumoniae* serotype 19F (Sp19, strain EF3030 provided by Dr. M. Lipsitch, Harvard School of Public Health, Boston, MA), LPS 0.5-1 µg/mL (Serotype 0111:B4, Sigma-Aldrich), or a mixture of 10 ng/mL TNF- α (Sigma-Aldrich #T7539) and 5 ng/mL IL-1β (R&D systems #401-ML) for pre-specified time points. Supernatants were collected for protein analysis and cells were collected in Trizol (Life Technologies) for RNA analysis.

### Human Alveolar Macrophages

Bronchoalveolar lavage (BAL) was performed on healthy, non-smoking volunteers. Following a standard informed consent protocol approved by Institutional Review Board of Boston University Medical Center, 240 mL sterile saline was instilled in 60 mL aliquots and recovered by gentle aspiration. Rates of recovery ranged from 160 to 210 ml. BAL fluid was strained through a single layer of gauze to remove large debris and centrifuged for 12 minutes at 450×*g*. BAL cells (BAL) were washed once in RPMI-1640. Differential counts were made on Dif-Quik and non-specific esterase (Sigma) stained cell preparations. Alveolar macrophages were isolated by adherence to plastic. Non-adherent cells were removed by vigorous washing and the remaining adherent cells average>98% viability as verified by trypan blue exclusion [51].

### Mice

Mice were maintained under pathogen free conditions in a barrier facility with access to food and water ad libitum. All experiments were performed using mice at 8–20 weeks of age. Experiments with non-transgenic mice were performed using C57BL/6 mice. Myeloid RelA deficiency was generated by interbreeding mice engineered to contain *loxP* sites flanking the critical exons 7–10 of RelA with LysM-cre^tg+^ mice, as described previously [52]. To create a conditional model of ZFP36L1 deletion, mice were engineered to contain *loxP* sites flanking exon 2 of the *Zfp36l1* gene resulting in a non-functional protein (*Zfp36l1^loxP/loxP^*). The ZFP36L1 flox animal model will be discussed in depth in a future report (PJB, manuscript in preparation). These mice were bred with mice that express Cre-recombinase driven by the endogenous lysozyme M promoter, which is active in cells of myeloid lineage (LysM-cre^tg+^) [53] to establish a colony of *LysM-cre^tg+^*/*Zfp36l1^loxP/loxP^* mice. Littermate controls *LysM-cre^tg−^*/*Zfp36l1^loxP/loxP^* were used for comparison.

### Experimental infection

For lung infections, mice were anesthetized by an intraperitoneal (i.p.) injection of ketamine (50 mg/kg) and xylazine (5 mg/kg). The tracheas were surgically exposed and cannulated using an angiocatheter that was directed to the left bronchus. A solution of bacteria (*E. coli or* Sp19) in sterile saline was instilled intratracheally (i.t.) [54]. These infections induce a reproducible lobar pneumonia and facilitate dissection of local events within infected lung tissues, but they does not incorporate nasopharyngeal colonization or other antecedent events that may be relevant to human patients with pneumonia. A target instillation of 0.5–1×10^6^ CFU of living bacteria was estimated by optical density and verified by quantifying serial dilutions grown on 5% sheep blood agar plates. To induce experimental septicemia, mice were infected with *E. coli* (10^5^ CFU) diluted in sterile saline instilled into the peritoneal cavity.

### Bronchoalveolar lavage

Bronchoalveolar lavage was performed after removal of the lungs *post mortem*. The trachea was cannulated with a 20-gauge blunted stainless steel catheter. Serial 1 ml lavage samples were taken x10 with ice-cold HBSS (no Ca^2+^ or Mg^2+^) containing 1 M HEPES, penicillin/streptomycin, and 0.5 M EDTA. The lavage fluid was centrifuged at 300×*g* for 5 minutes at 4°C, and cell pellets (>99% alveolar macrophages) were preserved. If used for primary culture, cell pellets were resuspended in serum-free RPMI1640 medium. The primary murine macrophages were seeded into 24 well plates and incubated overnight at 37°C in a humidified atmosphere containing 5% CO_2_. The cells were stimulated with 0.5–1 ug/ml LPS (Serotype 0111:B4, Sigma) or *E. coli*, 10^6^ CFU for pre-specified time points. Supernatants were collected for protein analysis and cells were collected in Trizol (Life Technologies) for RNA analysis. For BAL cytokine measurement, the first 1 milliliter lavage sample was saved separately for protein analysis. BAL cell counts were performed as previously described [52], [55], with cytocentrifuged slides stained with the Diff-Quick staining kit (Dade Behring) after counting suspended cells using a hemocytometer.

### Protein measurements

For immunoblot analysis, cells and lung tissue were snap frozen and protein was extracted from cell lysates. Protein concentrations were quantified using a bicinchoninic acid assay (Sigma-Aldrich), and Western blotting was performed using the NuPAGE gel system (Novex by Life Technologies). Membranes were probed using antibodies from Cell Signaling Technology against ZFP36L1 and ZFP36L1 (#2119S) and pan-actin (#4968S) as well as antibodies against ZFP36 (Sigma Aldrich #T5327), and IκB-α (Santa Cruz #SC-371). Primary antibodies were detected using an HRP-conjugated anti-rabbit antibody (Cell Signaling Technology #7074S), which was visualized using the ECLPlus Western Blotting Detection System (GE Healthcare). To determine cytokine content in supernatants of cell cultures, serum, and lung lavages, ELISA was performed. For whole lung cytokine analysis, lungs were homogenized in 5 ml sterile water with lung lysis buffer containing protease inhibitor (Roche #11849300) using the Next Advance Bullet Blender system. Homogenates were incubated on ice for 30 minutes, centrifuged, and supernatants were collected. ELISA was performed for TNF- α, and IL-6 using DuoSet ELISA Development kits (R&D Systems).

### Quantitative RT-PCR

Real-time RT-PCR was performed on 2–25 ng RNA using the Applied Biosystems TaqMan RNA-to-C_T_ 1-Step Kit, and either a StepOnePlus Real-Time PCR detection system (Applied Biosystems) or a C1000 Thermal Cycler system (Bio-Rad). For each sample, fold induction was normalized to the content of 18 s rRNA and expressed as fold-induction relative to a control group. The primers and TaqMan probe sets were designed with the CLC Genomics Workbench software (CLC bio) with the following sequences: Zfp36l1 forward 5′ GCTTTCGAGACCGCTCTTTCT 3′, reverse 5′ GGCACTTGTCCCCGTACTT 3′ and probe 5′ CAACTCCAGCCGCTACAAGACGG 3′; Zfp36 forward 5′ CGAGAGCCTCCAGTCGATGAG 3′, reverse 5′ GGATGGAGTCCGAGTTTATGTTCCAA 3′, and probe 5′ CCGACCACGGAGGAACCGAATCCCT 3′. IL-10 forward 5′ GCCCAGAAATCAAGGAGCATTTG 3′, reverse 5′ TTCACAGGGGAGAAATCGATGAC 3′ and probe 5′ AGCCGCATCCTGAGGCTGTTCAGC 3′ The TaqMan primer and probe sets for IL-6, TNF- α, IL-1β and 18 s rRNA have already been described [56], [57].

### Alveolar macrophage cell sorting

Mice were instilled with 1×10^6^ CFU of *E. coli* i.t. and sacrificed 0, 3, 6 or 24 hours post infection. Bronchoalveolar lavages were performed using ice-cold lavage buffer [HBSS (Life Technologies), 2.7 mM EDTA disodium salt solution (Sigma-Aldrich), 20 mM HEPES, 100 U/ml Pen-Strep]. Alveolar macrophages, defined as CD45^+^/7AAD^−^/F4/80^+^/Ly6G^−^, were sorted using a BD FACS Aria II. The following antibodies were used: CD45-APC clone 30-F11, Ly6G-APC/Cy7 clone 1A8, F4/80-PE eFluor 610 clone BM8. All antibodies, including 7AAD viability staining solution were purchased from Biolegend, except F4/80, which is from eBioscience. Cells were collected in PBS containing 1% BSA, centrifuged at 300×g for 5 minutes at 4°C and then resuspended in Trizol reagent. RNA was isolated following the manufacturer's protocol and qRT-PCR was performed as described above.

### RNA stability assay

Primary murine macrophages were collected from *LysM-cre^tg+^*/*Zfp36l1^loxP/loxP^, or LysM-cre^tg−^*/*Zfp36l1^loxP/loxP^* littermate controls, pooled from seven mice with respect to genotype, and cultured as above. RNA stability was measured by Actinomycin D blockade as previously described [30], [58]. Briefly, macrophages were stimulated with 1 µg/ml of LPS (Biolegend) for 2 hours. The LPS containing media was removed, and replaced with fresh medium containing 5 µg/ml of Actinomycin D (Sigma). RNA was collected by Trizol (Life Technologies) at the indicated time-points using the manufacturers instructions and quantitated by qRT-PCR as described above.

### Lung Histology and Immunohistochemistry

After euthanization, the heart was ligated to maintain pulmonary blood volume. Lungs were removed, instilled with 4% paraformaldehyde for fixation, and embedded in paraffin. For histology, lung sections were stained with Hematoxylin and Eosin. For immunohistochemical analysis, the lung sections were deparaffinized and subjected to antigen retrieval using Target Retrieval Solution (Dako). The slides were incubated with an antibody against ZFP36L1 (TISB Ab-92 Assay Biotech #B1184), ZFP36 (Sigma Aldrich #T5327), or a non-targeting antibody against rabbit IgG (Santa Cruz #SC-2027). Primary antibodies were then detected using ImmPRESS Anti-Rabbit Ig (peroxidase) Polymer Detection Kit (Vector Laboratories) and ImmPACT DAB Peroxidase Substrate (Vector Laboratories) and stained with Hemotoxylin and Eosin.

### Host defense and lung injury

To determine bacterial burden, whole lungs, livers, and spleens were homogenized in 5 ml sterile water using the Next Advance Bullet Blender system. Homogenates, BAL fluid, and blood samples were serially diluted and plated on 5% sheep blood agar plates. Plates were incubated overnight at 37°C in a humidified atmosphere containing 5% CO_2_. Colonies were counted and expressed as CFU. As a measure of lung injury, we assayed total protein concentration from the first 1 ml of lung lavage (described above) and quantified total airspace protein content using a bicinchoninic acid assay (Sigma-Aldrich).

### Statistical analyses

Statistical analyses were performed using GraphPad Prism (GraphPad Software). Data were presented as means +/− standard error. Real-time RT-PCR data were calculated as fold-induction and presented as geometric means +/− geometric standard error. Sets containing two groups of data were analyzed using a 2-tailed Student's *t* test or Mann-Whitney *U* test. Sets with more than two groups of data were analyzed using a 2-way ANOVA followed by a Bonferroni's post-test. Differences were considered statistically significant if p<0.05.

## Results

### ZFP36L1 protein expression is induced during infection

Given the physiologic importance of macrophage ZFP36 production in the negative regulation of cytokine production [15], [39], [52], we first investigated Zfp36l1 and Zfp36 expression in macrophages stimulated by bacteria or bacterial products *in vitro*. Alveolar macrophage-derived cells (MH-S cell line) in culture were stimulated with a gram-negative *Escherichia coli* (*E. coli*) or gram-positive bacteria *Streptococcus pneumoniae* serotypes 3 or 19 (respectively, Sp3 and Sp19), or LPS or a combination of two inflammatory cytokines, TNF- α and IL-1β. Our data show that both ZFP36L1 and ZFP36 protein were present at low levels at baseline and that both the gram-negative stimuli and one of the gram-positive stimuli (Sp19), as well as the pro-inflammatory cytokines resulted in increased production of these proteins (Figure 1B). During an infection time course, ZFP36L1 protein peaked at 2 hours and decreased to baseline between 6 and 24 hours (Figure 1C). *E. coli* also induced ZFP36L1 production in cultured primary murine and human alveolar macrophages at 2 hours (Figure 1D,E). In contrast to ZFP36L1, ZFP36L2 was not detected in cultured murine alveolar macrophages, and was present but not induced by *E. coli* in cultured human macrophages (Figure 1E and data not shown).

To determine whether macrophage ZFP36L1 production is induced similarly *in vivo*, C57BL/6 mice were infected with either *E. coli* or Sp19 as representative gram-negative and gram-positive infections. Alveolar macrophages were collected 0, 2 or 6 hours after infection and RNA was measured. At these early time points post infection, very low numbers of neutrophils had emigrated into the airspaces allowing for>95% purity in macrophage isolation via bronchoalveolar lavage. Similar to the *in vitro* results, ZFP36L1 and ZFP36 expression was induced by *E. coli*, although induction was highest 6 hours post infection (Data not shown). These results suggested that the optimal time point to investigate macrophage-dependent activity of ZFP36L1 would be between 2 and 6 hours.

**Figure 2:**
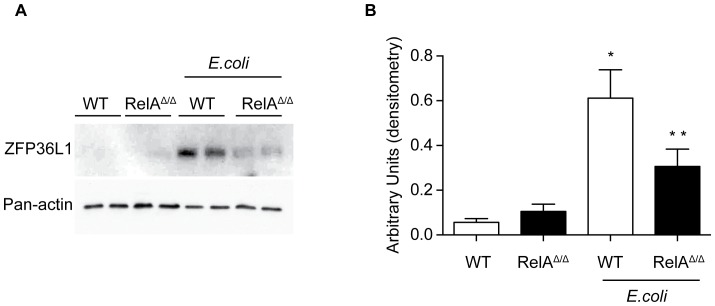
ZFP36L1 induction is partially dependent on NF-κB RelA. (A) Primary alveolar macrophages were obtained from control mice (WT) or mice with a myeloid specific deficiency of RelA (RelA^Δ/Δ^) and stimulated in culture with *E. coli* for 3 hours. (B) Densitometric analysis of immunoblot results from (A).

With the finding that ZFP36L1 is induced during infection in alveolar macrophages, we next sought to evaluate whether NF-κB signaling pathway plays a role in the rapid induction of ZFP36L1. Once bacteria are detected by pattern recognition receptors, cellular NF-κB RelA is critical to host defense, inducing multiple pro-inflammatory cytokines [52], [59], [60]. To determine whether ZFP36L1 could be induced by RelA *in vivo*, we obtained primary alveolar macrophages from mice with and without myeloid deficiency of RelA (LysM-cre^tg+^/RelA^loxP/loxP^) and infected them with *E. coli* for 3 hours (Figure 2A,B). We found that ZFP36L1 induction was reduced in the absence of RelA, suggesting that ZFP36L1 induction is at least partially dependent on myeloid NF-κB RelA activation.

### Mouse model of myeloid ZFP36L1 deficiency

**Figure 3:**
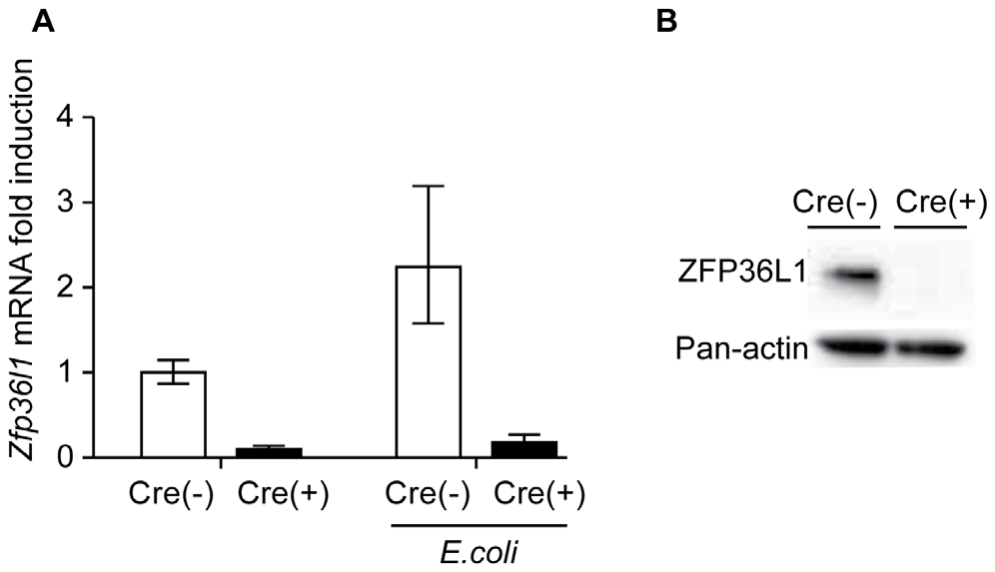
Mouse model of myeloid ZFP36L1 deficiency. (A) qRT-PCR analysis was used to quantitate *Zfp36l1* mRNA levels in alveolar macrophages obtained from Cre (−), or Cre (+) mice 0 or 3 hours after i.t. infection with *E. coli* (n = 3–4). Results indicate fold change relative to uninfected Cre (−).(B) Immunoblot analysis of murine alveolar macrophages from Cre (−), or Cre (+) mice stimulated *in vitro* with LPS for 2 hours (n = 3).

To evaluate whether ZFP36L1 plays a physiological role in limiting cytokine expression during infection we sought to measure cytokine levels *in vivo* in mice with a deficiency of ZFP36L1. In contrast to ZFP36 knockout, constitutive ZFP36L1 knockout results in embryonic death [41], therefore it was necessary to engineer mice containing *loxP* sites flanking the second exon of *Zfp36l1* that would be amenable to conditional deletion. Given that myeloid cells appear to be a major cell type responsible for ZFP36's regulation of cytokine expression, we chose to breed mice with a myeloid-specific *Zfp36l1* deletion. This was achieved by interbreeding C57BL/6 mice containing Cre-recombinase driven by a Lysozyme M promoter (LysM-cre) with a mouse line engineered to contain a floxed *Zfp36l1* gene. The LysM-cre model has been shown to result in Cre expression predominantly in myeloid cells including monocytes, mature macrophages, granulocytes and a limited amount of dendritic cells, with an 83–98% deletion efficiency in mature macrophages [53]. Excision of this 2^nd^ exon results in a truncated non-functional protein. *Zfp36l1* deficiency was confirmed by RNA and protein measurements from alveolar macrophages (Figure 3A,B).

### Basal expression of ZFP36L1 does not repress cytokine production

**Figure 4:**
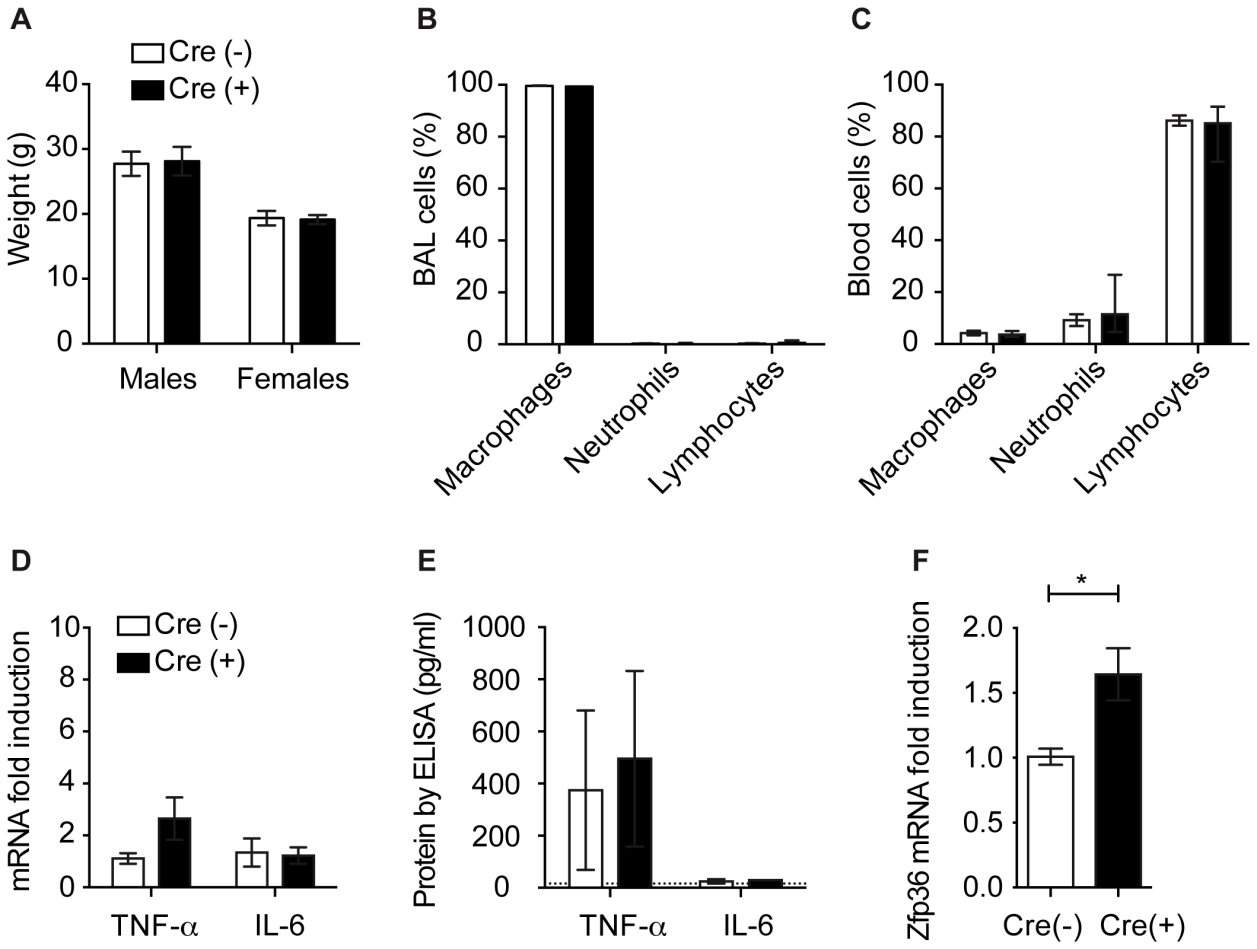
Myeloid ZFP36L1 deficiency does not result in spontaneous immunopathology. (A) Average weight (n = 12–16) of 8–20 week old mice measured at time of experimentation (B) BAL differential cell counts (n = 7,7) or (C) blood cell counts (n = 7,7) in mice with and without myeloid deficiency of ZFP36L1 (D) TNF- α and IL-6 mRNA in alveolar macrophages obtained from uninfected mice (n = 10,10) (E) TNF-α and IL-6 protein levels measured by ELISA from BAL fluid of uninfected mice (n = 6,6) (F) qRT-PCR analysis of *Zfp36* mRNA from alveolar macrophages collected from uninfected mice (n = 6,6 p<0.05). Results indicate fold change relative to Cre (−).

One of the most salient phenotypes of the ZFP36 knockout mouse model is that the mice develop spontaneous inflammatory pathology [29], [52]. This was a prominent concern from the outset of the studies, given such close homology between the Zfp36 family members, so we first sought to determine whether our mice with myeloid ZFP36L1 deficiency also exhibited elevated inflammatory markers at baseline. We found no differences between our young adult mice with and without myeloid ZFP36L1 deficiency in total body weight (Figure 4A) or overall appearance of the mice. Lung histology appeared grossly similar, and there were no differences in BAL or blood white blood cell (WBC) differential counts (Figure 4B,C). Relative levels of mRNA for select macrophage-produced cytokines from lavaged cells were similar between the two groups (Figure 4D), as were protein levels in the BAL fluid (Figure 4E). Interestingly, the one difference that we identified between groups was that the mice deficient in ZFP36L1 had heightened levels of ZFP36 mRNA in the lavaged macrophages (Figure 4F). These data indicated that myeloid ZFP36L1 is not involved in basal regulation of inflammation.

### Macrophage ZFP36L1 is not required for the early cytokine response to pneumonia

**Figure 5:**
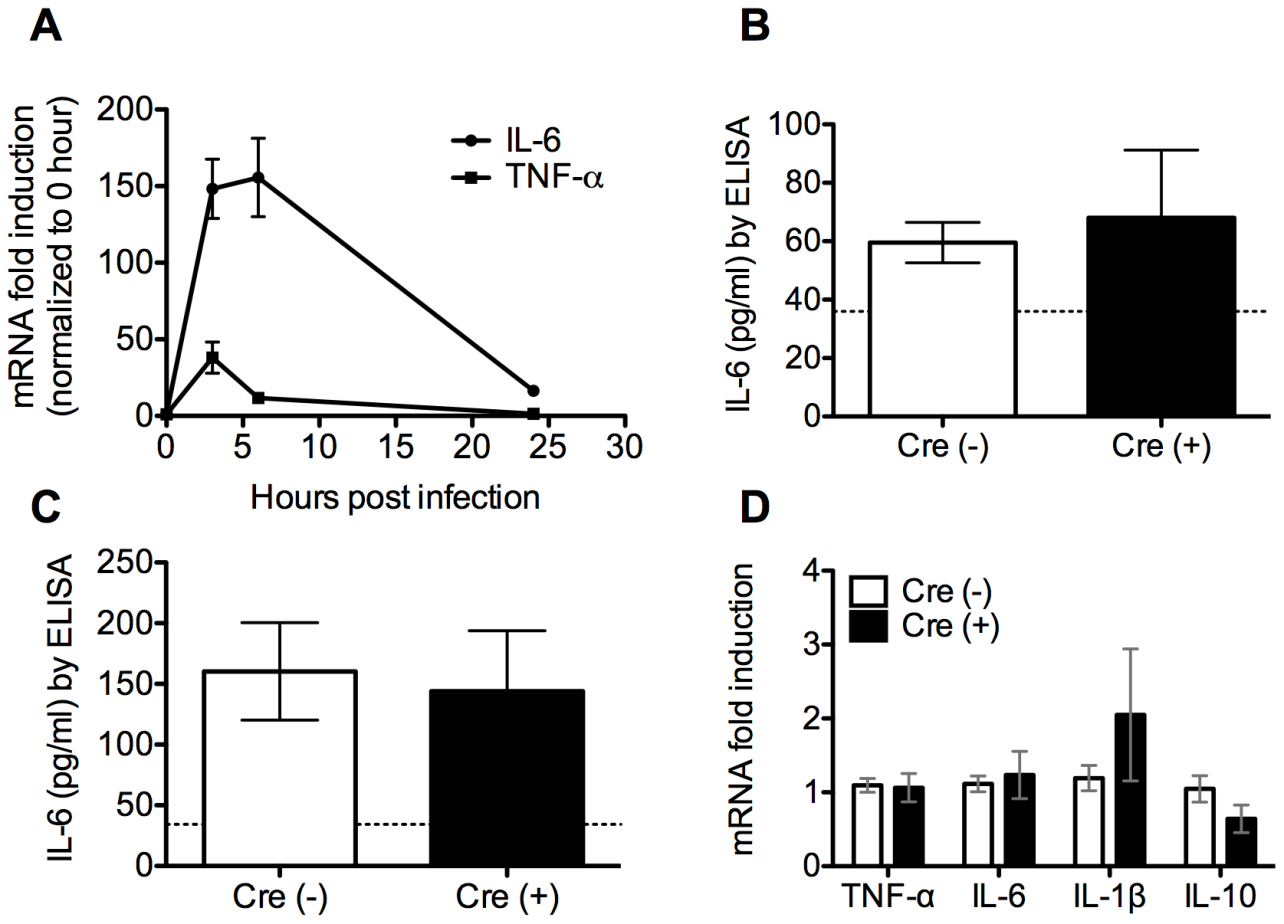
Macrophage ZFP36L1 is not required for regulating the early cytokine response during pneumonia. (A) qRT-PCR of alveolar macrophage RNA collected from C57BL/6 mice infected with *E. coli*. Results indicate fold change relative to uninfected with respect to genotype. Mice with and without myeloid deficiency of ZFP36L1 were infected i.t. with *E. coli*. IL-6 protein levels were measured by ELISA in (B) serum (n = 13,10) and (C) BAL fluid (n = 13,10) (D) qRT-PCR analysis of TNF- α, IL-6, (n = 20–22) IL-1β, (n = 13,12) and IL-10 (n = 4,4) mRNA from alveolar macrophages collected by BAL 3 hours post infection with *E. coli*. Results indicate fold change relative to Cre (−).

**Figure 6:**
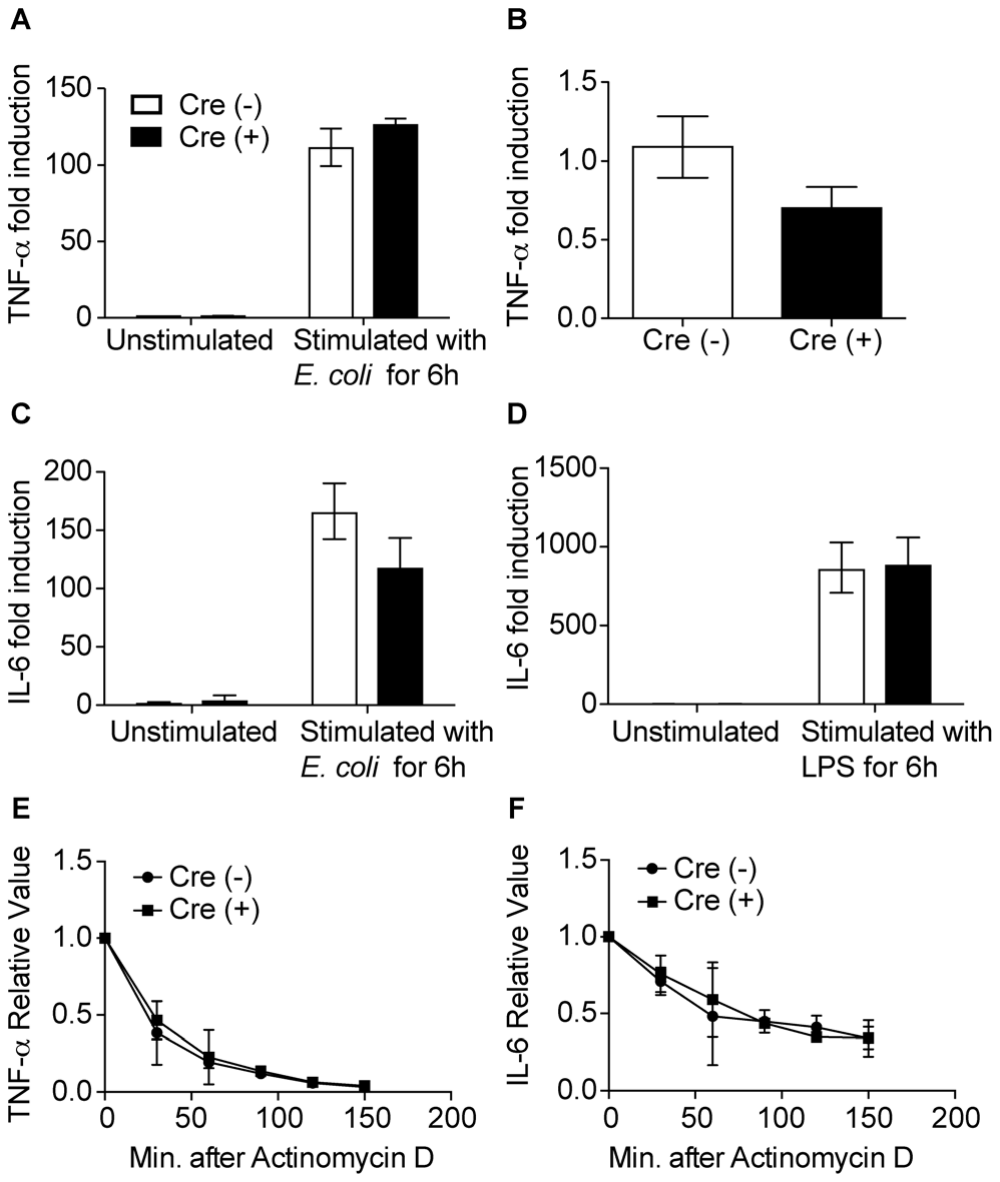
ZFP36L1 deficient macrophages are capable of maximal cytokine expression. qRT-PCR analysis of primary alveolar macrophages collected from Cre (−) or Cre (+) mice, and stimulated with (A,F) vehicle or *E. coli* for 6 hours (n = 3) or (B,D) LPS (n = 7,5). Results indicate fold change relative to unstimulated Cre (−) (for A,F) or Cre (−) (B,D) (E) Relative TNF- α and (F) IL-6 half lives in Cre(−) or Cre (+) alveolar macrophages after 2 hours of LPS stimulation followed by transcriptional blockade by Actinomycin D.

Given a lack of basal inflammation in the myeloid-deficient ZFP36L1 mice, we proceeded to determine whether these mutant mice would exhibit enhanced cytokine production at early time points after an infectious challenge. We utilized a clinically relevant, and previously described mouse model of gram-negative pneumonia [57], [61]. *E. coli* is known to cause pneumonia in ventilated patients and the elderly, and in mice generates a titratable infection, ranging from self-limiting to 100 percent mortality based on dose [62]–[66]. In order to determine an optimal time point with maximal cytokine expression, we infected C57BL/6 mice with a severe but sub-lethal dose of *E. coli*, and sorted airspace macrophages at several time points of infection. Results indicated that peak TNF- α and substantial IL-6 expression at 3 hours post infection (Figure 5A). We next infected Cre (−) and Cre (+) mice with the same dose of *E. coli* and contrary to our hypothesis, we were surprised to find no difference in levels of select cytokine protein levels in the BAL fluid (Figure 5B,C) or mRNA in the lavaged cells (Figure 5D) at 3 hours after infection. Consistent with this finding, primary alveolar macrophages from myeloid ZFP36L1 deficient mice and controls stimulated in culture with *E. coli* or LPS had similar levels of TNF-α and IL-6 mRNA (Figure 6A-D). No difference was seen in mRNA half life for TNF- α or IL-6 in cultured alveolar macrophages stimulated with LPS (Figure 6E,F). Further, serum levels of TNF-α and IL-6 and did not differ between either genotype (Data not shown). In summary, in contrast to published data showing that inflammatory mediators such as TNF- α, IL-3, and GM-CSF are regulated by ZFP36L1 in cultured cells [18], [49], [50], our data demonstrate that in primary macrophages ZFP36L1 does not regulate expression of any of the cytokines we assayed.

### Myeloid ZFP36L1 does not regulate pulmonary host defense or lung injury

**Figure 7:**
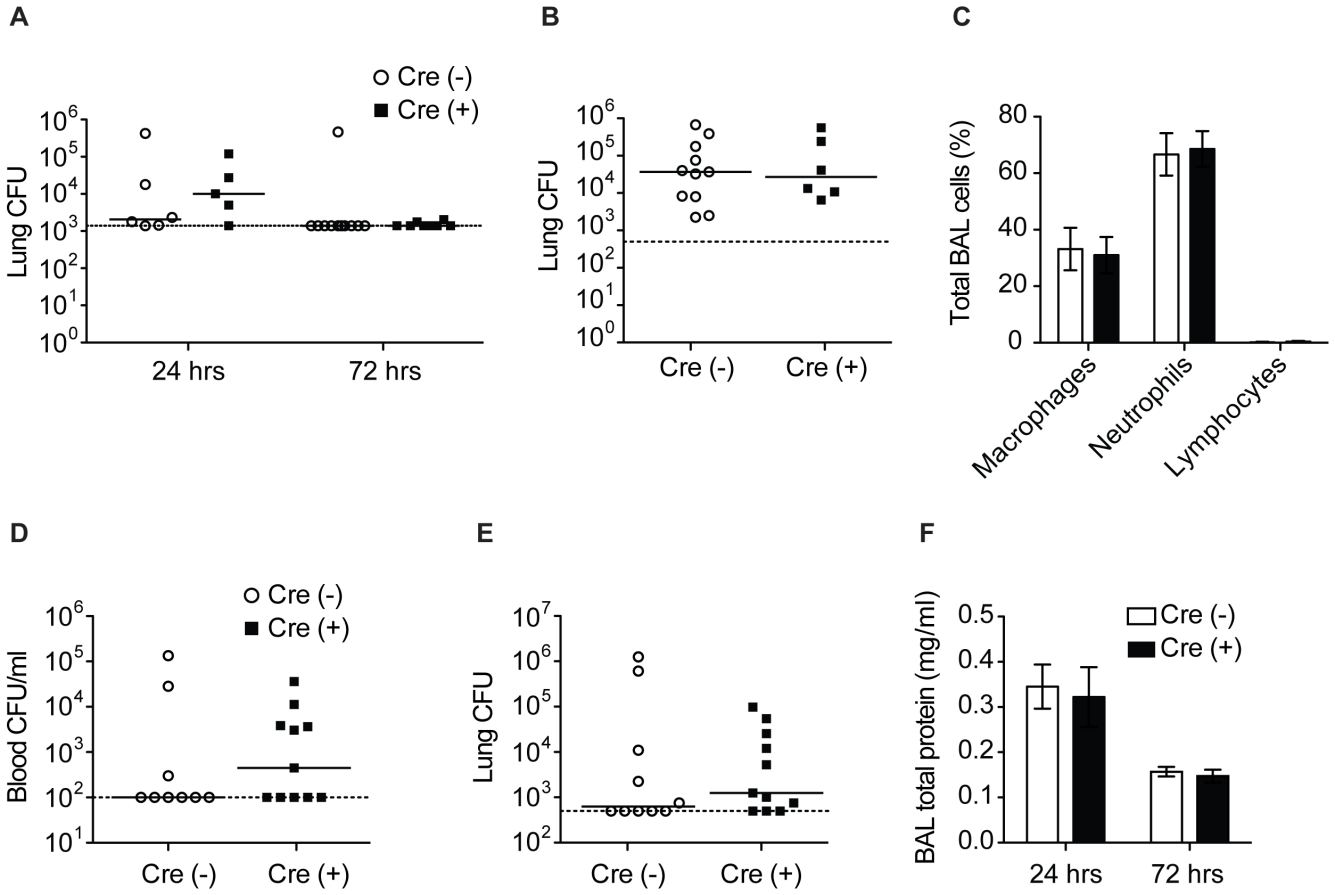
Myeloid ZFP36L1 does not regulate pulmonary host defense or lung injury. Lung bacterial burden from Cre(−) or Cre(+) mice after i.t. instillation of (A) *E. coli* or (B) after 24 hours *S. pneumoniae* serotype 19 infection. (C) BAL differential cell counts in Cre (−) and Cre (+) mice (n = 6,5) after 24 hours i.t. *E coli*. Bacterial burden in (D) blood and (E) lung 24 hours after intraperitoneal infection with *E. coli*. (F) Total protein concentration in BAL fluid as a measurement of lung injury 24 or 72 hours after infection with *E. coli* (n = 5–8).

As a more dynamic and complete measure of host defense, we sought to evaluate bacterial burden and lung injury during pneumonia. Mice were intratracheally infected with either *E*. coli or Sp19 to cause a lobar pneumonia, and bacterial burdens were examined at 24 and 72 hours. The *E. coli* model is described above. Our laboratory has previously described the Sp19 model, involving a focal pneumonia in which pulmonary antibacterial defenses are strongly dependent upon effective inflammatory responses, as evidenced by defective immunity and excessive bacterial outgrowth in mice with whole animal lack of RelA [59], lack of myeloid RelA [52], lack of alveolar epithelial RelA [67], disruption of TNF- α and IL-1 signaling [61], or ablation of the acute phase response [68]. Similar to the results of the cytokine data, no differences were observed in indicators of host defense between the mice with and without myeloid deficiency of ZFP36L1 under either infection. Markers of bacterial infection, as measured by lung bacterial burden, occurrence of bacteremia, and degree of weight loss (Figure 7A-B and data not shown) were all statistically equivalent. Moreover, we observed no differences in BAL differential leukocyte counts (Figure 7C) or cytokine levels by RNA and protein analysis (data not shown). As an indicator of lung injury, we measured total BAL protein levels after 24 and 72 h after *E. coli* infection and found that, similar to host defense, there was no difference between genotypes (Figure 7F).

To determine whether there were differences in cytokine expression or bacterial clearance after infection not localized to the lung, mice were infected intraperitoneally with *E. coli* to cause a systemic sepsis syndrome. There were no differences between genotypes in bacterial burdens from blood, lung, liver or spleen, and there were no differences in serum TNF- α or IL-6 levels (Figures 7D-E and data not shown). In summary, our data indicated that macrophage ZFP36L1 does not influence antibacterial host defense during pneumonia or sepsis.

### Multiple cellular sources of ZFP36L1 expression in lungs

**Figure 8:**
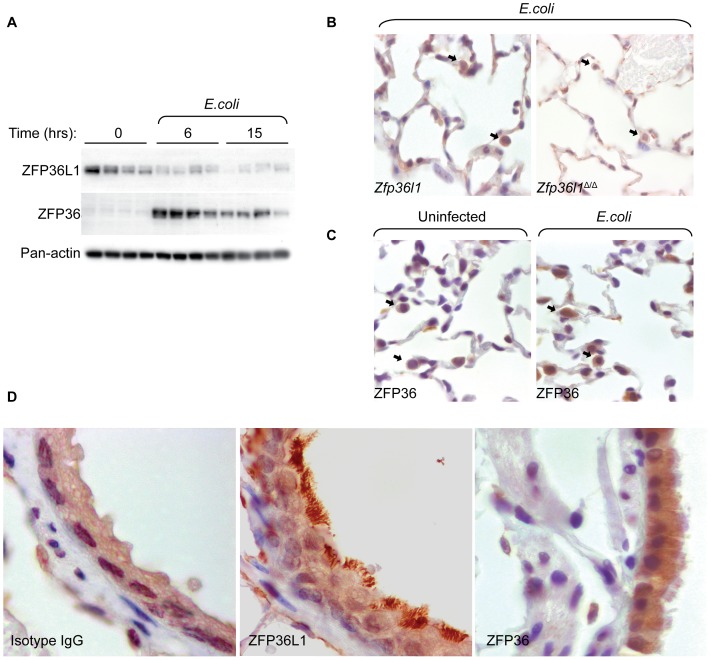
Evidence for multiple cellular sources of ZFP36L1 expression in lungs. (A) Immunoblot from whole lung homogenate from C57BL/6 mice 0, 6 and 15 hours after infection with *E. coli*. (B) Immunohistochemical staining of lungs from Cre (−) (left panel) and Cre (+) (right panel) mice showing ZFP36L1 expression in macrophages (arrows). (C) Staining of lungs from C57BL/6 mice uninfected (left panel) and 2 hours after infection with *E. coli* (right panel) demonstrating ZFP36 expression in macrophages (arrows). (D) Ciliated epithelium exhibits strong ZFP36L1 staining in uninfected animals (middle panel) which is not seen with IgG isotype control (left panel) or ZFP36 staining (right panel).

Our unanticipated data suggested that myeloid-specific deletion of ZFP36L1 exhibited no impact on select cytokine levels, markers of lung defense, or inflammatory lung injury during pneumonia and sepsis. As a potential reason for this clear lack of phenotype, we sought to determine ZFP36L1 expression patterns in the whole lung to determine whether other cells that contribute to an integrated host response to infection are also sources of ZFP36L1. We examined whole lung expression of ZFP36 and ZFP36L1 at 0, 6 and 15 hours after *E. coli* pneumonia. As expected, ZFP36 expression in the whole lung is similar to that in macrophages, expressed at low levels at baseline and strongly induced at 6 and 15 hours (Figure 8A). Unlike ZFP36 expression, ZFP36L1 levels are maximal under normal conditions and reduced during an infection time course (Figure 8A). It appears that although ZFP36L1 is induced in macrophages, that induction is completely overwhelmed by extra-myeloid source of ZFP36L1 expression. To determine what other cell types express ZFP36L1 in the lung, we performed immunohistochemical staining of fixed lungs uninfected or infected with *E. coli* pneumonia. In addition to staining in macrophages, epithelial cells exhibited prominent staining for ZFP36L1, both in the alveoli and in ciliated cells lining the conducting airways (Figure 8B,D). Although ZFP36 staining was present in other cell types, it was predominantly present in alveolar macrophages (Figure 8C) and did not show prominent staining in epithelial cells (Figure 8C,D). These data demonstrate that unlike ZFP36, ZFP36L1 expression is reduced in the whole lung during pneumonia and expressed in extra-myeloid cells in the lung.

## Discussion

In this study, we demonstrated that myeloid deficiency of ZFP36L1 exhibited no observable impact on innate host defense or cytokine expression during acute bacterial pneumonia. Because myeloid-deficient ZFP36 mice exhibit a prominent spontaneous inflammatory disorder [29], [52], we first investigated ZFP36L1 mutant mice in the absence of infection to determine whether they have heightened levels of inflammation at baseline. In contrast to the ZFP36-null mice, myeloid ZFP36L1 deficiency exhibited no detectable differences in lung cytokine levels, inflammatory cell differential counts, or gross lung histology. Interestingly, the one difference we identified was that ZFP36 mRNA levels were increased in lavaged macrophages. This finding indicates that ZFP36 may be increased to functionally compensate for the lack of ZFP36L1, supporting the notion that the Zfp36 family members have some overlapping functions. Although, to our knowledge, ZFP36L1 levels have not been measured in the ZFP36 deficient mice it is clear that even if it were increased to compensate for the lack of ZFP36, it is insufficient to limit cytokine over-production and inflammatory damage.

Despite the lack of increased cytokines in the lung at baseline in the ZFP36L1 deficient mice, we hypothesized that we would observe a difference in cytokine expression under stimulated conditions such as early after infection. Given that ZFP36L1 is present only in low levels at baseline in alveolar macrophages and is rapidly induced in response to bacteria with a peak expression *in vivo* between 2 and 6 hours, we measured cytokine levels as well as inflammatory cell recruitment to the lung at early time points after infection. We were surprised to find that there were no detectable differences in select macrophage produced cytokine in the BAL fluid, including TNF- α, which is overproduced in the absence of myeloid ZFP36. There was also no alteration in differential leukocyte counts at baseline or at 3 hours after infection, and the two groups had similar neutrophil recruitment demonstrated after 6 hours of infection. Consistent with this finding, we also did not detect any differences in select cytokine production in cultured murine alveolar macrophages 6 hours after stimulation with *E. coli* or LPS (data not shown). Of note, measurements of ZFP36 mRNA 3 hours after infection were no longer different between the groups. While these findings were surprising, we acknowledge that measurements at isolated time points after infection are limited in their ability to detect alterations in such a dynamic and rapidly-changing response.

Given the limitations of measurements and isolated time points, to best determine whether myeloid ZFP36L1 is important for the inflammatory response of the lung and for maintaining the balance between sufficient inflammation to prevent pneumonia but not enough to cause excessive lung injury, we next evaluated outcomes of pneumonia at longer time points. Contrary to our hypothesis, we found no differences in markers of bacterial clearance, severity of illness or lung injury in either a gram positive (Sp19) or a gram negative (*E. coli*) lung infection. Further, there were no differences in these markers after a systemic infection inducing a global sepsis response *E. coli*. This is particularly notable, as mice with myeloid deficiency of ZFP36 show extreme susceptibility to sepsis induced by LPS [52]. Contrary to our hypothesis, this lack of an innate immune phenotype indicated that either ZFP36L1 does not play a role in an integrated host defense, the presence of ZFP36 and/or ZFP36L2 can compensate for the loss of ZFP36L1 as has been recently shown in T cells [44], or that ZFP36L1 expression in other innate immune cells outside of the myeloid lineage compensate for macrophage ZFP36L1 deficiency.

In summary, it was unanticipated to find that myeloid deficiency of ZFP36L1 does not appear to be important for the lung's inflammatory response to pneumonia. Previous work has suggested that myeloid cells, specifically macrophages, are the primary source of Zfp36 proteins for the regulation of inflammation. Interestingly, recently published work using the same LysM-cre model to create myeloid deficiency of ZFP36 demonstrates that although the mice do have elevated levels of TNF-α and signs of systemic inflammation, the phenotype is mild in comparison with the systemic inflammatory syndrome that develops in full ZFP36 knockout mice. These results in combination with our findings led us to explore whether other cells in the lung are significant sources of ZFP36L1 production, which may act to compensate for the myeloid deficiency. Our immunohistochemical staining suggests that ZFP36L1 may be expressed in multiple cell types in the lung, with strong staining in lung epithelial cells. This staining was apparent both in alveolar lining cells and in the conducting airway ciliated cells. In comparison, while ZFP36 staining also demonstrates that there may be additional cell types in the lung expressing ZFP36, overall staining appears to be more localized to macrophages. This together with our findings that ZFP36L1 expression in the whole lung decreases in response to infection, while its expression in macrophages is increased, suggests that other cell types may be a more important source of ZFP36L1 in the lung.

The lack of importance of myeloid ZFP36L1 in the lung's defense against pneumonia is surprising and raises still more questions about the Zfp36 family members and their role in post-transcriptional modification of the inflammatory response. What is the significance of differential expression of family members in different cell types, and what cells in addition to macrophages are important for the phenotypes identified in deficient mice? Also, what are the interactions between the Zfp36 family members? Our findings raise the possibility that ZFP36 may be increased to help compensate for lack of myeloid ZFP36L1 and thus prevent noticeable alterations in the lung's inflammatory response, it does not appear that ZFP36L1 is able to compensate similarly in myeloid ZFP36 deficiency. Other areas of future study include further evaluation of phosphorylation and differential activation status of these proteins and their interactions with other post-transcriptional modulators of immune function. Although understanding of post-transcriptional modification of immune function is important and has implications for human disease, it is clear that the mechanisms in place to fine-tune and regulate the response are complex.
